# A Novel Adsorbent Albite Modified with Cetylpyridinium Chloride for Efficient Removal of Zearalenone

**DOI:** 10.3390/toxins11110674

**Published:** 2019-11-18

**Authors:** Wei Zhang, Shihua Zhang, Jingjing Wang, Jiawen Dong, Baojing Cheng, Li Xu, Anshan Shan

**Affiliations:** Institute of Animal Nutrition, Northeast Agricultural University, Harbin 150030, China; zhangwei910315@hotmail.com (W.Z.); zhangshihua94@hotmail.com (S.Z.); JF_JING@hotmail.com (J.W.); dongjiawen2018@outlook.com (J.D.); chengbaojing@neau.edu.cn (B.C.)

**Keywords:** adsorption, zearalenone, cationic surfactant, cetylpyridinium chloride, albite

## Abstract

Zearalenone (ZEN) is a non-steroidal estrogenic mycotoxin and constitutes a potential health threat to humans and livestock. This study aimed to explore the potential of albite modified by the cationic surfactant cetylpyridinium chloride (CPC) as ZEN adsorbent. The organoalbite (OA) was characterized by SEM analysis, XRD analysis, FTIR spectroscopy, thermal analysis, and BET gas sorption measurement. In vitro adsorption of ZEN by OA was carried out by simulating the pH conditions of the gastrointestinal tract. The characterization results showed that the surface of OA changed from hydrophilic to hydrophobic after modification. Adsorption kinetic studies showed that ZEN adsorption behavior of OA occurred by chemisorption. The equilibrium adsorption data fitted well with the Langmuir isotherm, indicating that the adsorption process of ZEN by OA was monolayer. The maximum adsorption capacity (q_m_) values of OA for ZEN were 10.580 and 9.287 mg/g at pH 7 and pH 3, respectively. In addition, OA had a low desorption rate (about 2%), and co-existing amino acids (i.e., Lys and Met), vitamins (i.e., VB_1_ and VE), and minerals (i.e., Fe^2+^ and Ca^2+^) did not affect the removal of ZEN. These results demonstrate that OA could be a promising mycotoxin adsorbent for removing the hydrophobic, weakly polar ZEN.

## 1. Introduction

Mycotoxins are toxic secondary metabolites produced by certain fungi that accumulate in maturing corn, cereals, and other food and feed crops [1]. Their presence in cereal crops not only causes economic losses, but also affects human health and animal productivity as a result of indirect exposure [2]. Zearalenone (ZEN), a common estrogenic mycotoxin produced by *Fusarium* fungi, demonstrates immunotoxic, genotoxic, hyperestrogenic, hematotoxic, carcinogenic, hepatotoxic, and teratogenic effects on a variety of mammalian species [2]. The major toxicity of ZEN and its metabolites is attributed to their estrogenic effects on reproductive organs, such as the ovary, uterus, and testis [3,4,5]. Consequently, searching for an effective method to remove mycotoxins is becoming one of the most urgent challenges.

Currently, the detoxification strategies for mycotoxins are mainly chemical, biological, and physical processes. Previous studies have shown that mycotoxins could be eliminated by chemicals such as H_2_O_2_, 1N aqueous citric acid, and ammonium hydroxide [6,7,8]. However, the reduction of nutritional value and palatability has restricted the popularization of this chemical method. Another attractive method is to degrade part of the mycotoxins with microorganisms or enzymes. Previous studies have shown that several microorganisms, such as *Bacillus*, *Pseudomonas,* and *Lactobacillus* species, could effectively degrade mycotoxins in vitro [9,10,11,12,13]. However, the application of this method is also limited by the complex environment in vivo and high cost. At present, the most feasible and economical detoxification method is the use of adsorbents for physical adsorption. Adsorbent as feed additive can reduce the absorption of mycotoxin by organism [14]. Many previous studies have reported that natural silicate minerals, such as montmorillonite, zeolite, and hydrated sodium calcium aluminosilicate, could effectively bind polar mycotoxin aflatoxin B_1_, reducing its toxicity [15,16]. Nevertheless, the binding efficiency of these hydrophilic negatively-charged silicate minerals to low polar and hydrophobic mycotoxins such as ZEN is low [17,18].

Albite is a sodium-rich mineral of the plagioclase feldspar group, which occurs widely in felsic rocks [19]. The framework structure is mainly composed of linked SiO_4_ and AlO_4_ tetrahedrons, which contain large vacancies, i.e., lattice positions [20]. This structure exhibits certain ion exchange properties and has distinct pore structure mineral characteristics. Some previous studies have suggested that albite shows effective adsorption of uranium (VI), gold (Au), and neptunium (Np) by the formation of secondary mineral phases at the albite surface [21,22,23,24]. However, to our knowledge, studies of albite used as a mycotoxin adsorbing agent have not been reported. In order to adsorb hydrophobic molecules with low polarity, long-chain cationic surfactants are usually used to substitute inorganic cations of non-metallic minerals, which change the surface properties from hydrophilic to hydrophobic [25]. Furthermore, the adsorption efficiency depends on the type and chain length of cationic surfactants [26].

In this work, a novel albite-based ZEN adsorbent was fabricated by using the cationic surfactant CPC as a modifier. SEM, XRD, FTIR, BET, and TG–DSC were employed for the characterization of the product. Batch adsorption experiments were carried out to explore the adsorption behavior and adsorption mechanism of ZEN on organoalbite (OA). This study provides the theoretical basis for the application of modified albite as a mycotoxin adsorbent in the removal of ZEN in feed.

## 2. Results and Discussion

### 2.1. Characterizations

#### 2.1.1. SEM Analysis

**Figure 1:**
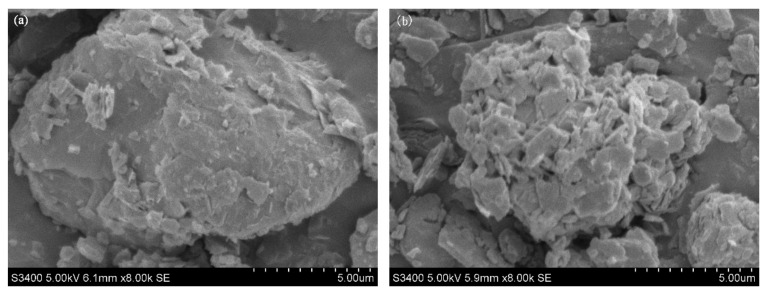
SEM images of natural albite (**a**) and the prepared organoalbite (**b**). Scale bar: 5.00 μm.

The morphology of albite before (NA) and after (OA) modification was characterized by SEM imaging. As shown in Figure 1, both NA and OA are large particles. The surface of NA was relatively flat and smooth; a regular and compact stacked lamellar structure was observed with thicker lamellae and fewer pores. After CPC treatment, the particle size and structure of OA were remarkably altered by forming more holes and cracks with more exfoliated, loose, and curled layers. The view from the side shows that there is a certain distance between the layers, accompanied by some sharp edges, and there are more fine particles between the layers. The surface morphology directly affects the adsorption capacity for heavy metal ions and organic pollutants. The structure of OA showed favorable channels and voids for adsorbing ZEN.

#### 2.1.2. XRD Analysis

**Figure 2:**
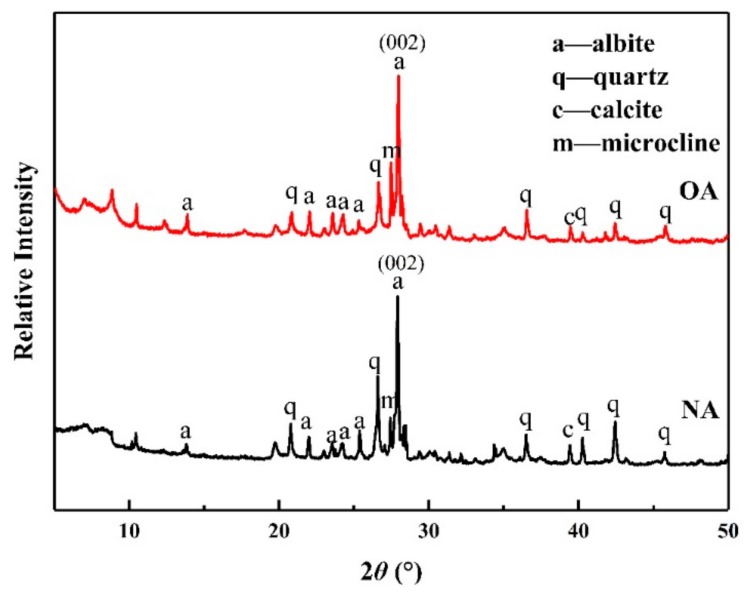
XRD pattern of natural albite (NA) and the prepared organoalbite (OA).

The crystal structures of the materials were characterized by XRD. In Figure 2, the XRD diagram demonstrates that the diffraction peak at 2θ = 27.93° was the typical diffraction peak of albite. The basal spacing of natural NA was 3.19 Å, indicating the 0 0 2 plane of albite. Moreover, it has been confirmed from the diffractograms that small amounts of quartz, calcite, and microcline can be found in NA and OA. The XRD patterns showed that the diffraction peaks of OA were the same as those of NA, which indicated that the introduction of CPC had no impact on the transformation of the crystal phase structures. However, a slight decrease in peak sharpness for the samples was observed after modification, indicating that the crystallinity changed slightly after the CPC treatment process. These findings suggest that the grafting sites mainly occur on the outer surface, but less between the interlayers, which might be due to the restriction of the NA molecular structure by the modifier entering the interlayer.

#### 2.1.3. FTIR Analysis

**Figure 3:**
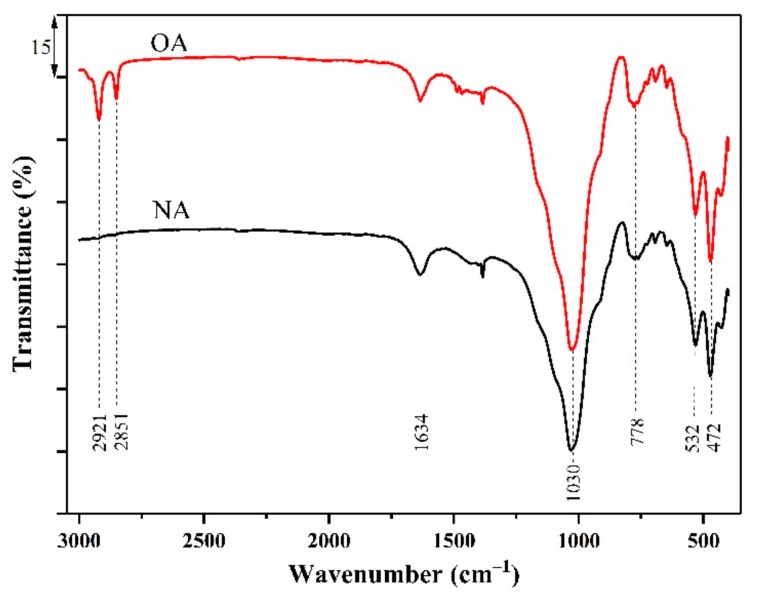
FTIR spectra of natural albite (NA) and the prepared organoalbite (OA).

The FTIR spectra of NA and OA are presented in Figure 3. The change in structural orderliness can be determined by the increase in intensity, number, and sharpness, as well as by the shifts in band frequencies of the peaks [19]. It is known that the main infrared characteristic peaks of albite occur at 400–1300 cm^−1^ [27]. In this range, there were four similar bands in the starting albite and organoalbite samples, which appeared at approximately 472, 532, 778, and 1030 cm^−1^. The two obvious peaks centered at 472 cm^−1^ and 532 cm^−1^, represented the bending vibration of Si–O–Si and the coupling between the O–Si–O deformation and the Na–O stretching, respectively [27,28]. The band at 778 cm^−1^ was attributed to Si–O–Si bending vibrations, while the Si–O symmetric bond stretching bands could be seen in the region of 900–1200 cm^−1^ centered at 1030 cm^−1^ [19,29]. Furthermore, the peak centered at 1634 cm^−1^ and 3453 cm^−1^ correspond to the bending and stretching vibrations of water molecules [17,30]. This meant that there were H-bonded hydroxyls and physically adsorbed water attached on the albite and organoalbite surfaces. However, the bands between 2800–3000 cm^−1^ associated with the symmetric and asymmetric CH_2_ stretching modes of the alkyl chain were particularly present in OA, indicating that surfactant molecules have successfully inserted NA [17,30].

#### 2.1.4. The Contents of Organic Carbon (C), Hydrogen (H), and Nitrogen (N)

The organic carbon contents of albite before and after modification with CPC were obtained from the elemental analysis (Table 1). After modification with CPC, the contents of C, H, and N were increased and reached 5.94%, 1.26%, and 0.34%, respectively. The genuine surfactant adsorbed on the surface of albite was calculated according to the N content. CPC-modified albite had 0.998 cation exchange capacity (CEC) of CPC, implying that almost all exchangeable cations were successfully loaded on the albite surface. It has been found that the modification of montmorillonite with cationic surfactants improved the adsorption of hydrophobic, weakly polar contaminant ZEN [18].

#### 2.1.5. Surface Area and Pore Size Distribution

**Figure 4:**
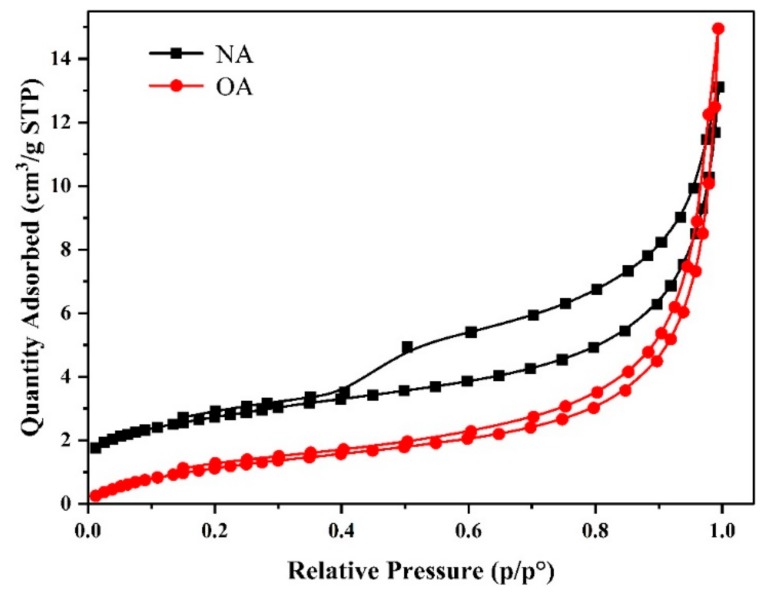
N_2_ adsorption–desorption isotherms of natural albite (NA) and the prepared organoalbite (OA).

The BET gas sorption measurements were conducted at −196 °C to demonstrate the presence of micropores and mesopores in the albite before and after modification. Figure 4 shows the N_2_ adsorption–desorption isotherms for NA and OA. According to the new IUPAC classification, the isotherms of both NA and OA exhibited type IV (a) sorption behaviors [31]. Moreover, a clear H3-type hysteresis loop was observed at the relative pressure range of 0.4–1.0, implying that the two samples have mesoporous structures [32].

The characteristic specific surface area, together with pore volume and size, were analyzed based on the BET and BJH methods (QSDFT simulation mode), and are listed in Table 2. The average pore diameters of NA and OA were both in range of 2–50 nm, indicating the presence of mesopores. This was in agreement with the results obtained from the nitrogen adsorption–desorption isotherms in Figure 4. The specific surface area of NA was 9.80 m^2^/g, the BJH pore volume was 0.02 cm^3^/g, and the average pore diameter was 7.92 nm. Compared with NA, smaller specific surface area (4.87 m^2^/g) and larger pore size (17.86 nm) were found on OA, which might be attributed to the loading of surfactant molecules [33]. These results demonstrate that the distribution and arrangement of CPC molecules on the surface control the efficiency of ZEN sorption by OA rather than the BET-N_2_ surface area, pore volume, and pore diameter [34].

#### 2.1.6. Surface Hydrophobicity

**Figure 5:**
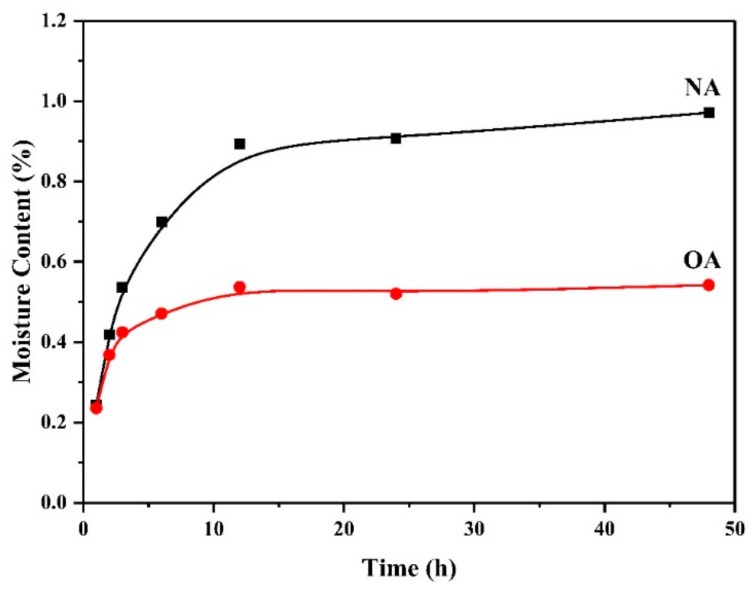
Moisture adsorption capacities of natural albite (NA) and the prepared organoalbite (OA).

The surface hydrophobicities of NA and OA are shown in Figure 5. NA and OA exhibited continuous mass gain within 12 h and then tended to be stable. The final moisture adsorption capacities dropped from 0.97% for NA to 0.54% for OA, which inferred that hydrophobicity of OA increased. This was beneficial to capture the nonpolar, hydrophobic molecule ZEN through hydrophobic interaction.

#### 2.1.7. Thermal Analysis

**Figure 6:**
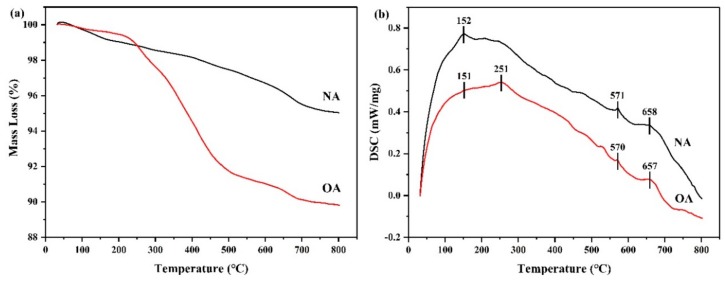
TG (**a**) and DSC (**b**) curves of natural albite (NA) and the prepared organoalbite (OA).

Thermal analysis (TG–DSC) was conducted to evaluate the thermal properties of albite samples before and after modification. The thermogravimetric (TG) and differential scanning calorimeter (DSC) curves of the NA and OA are presented in Figure 6a,b, while their mass losses in different temperature regions are shown in Table 3. The mass loss was divided into three parts: (1) Mass loss observed in the temperature range of 25 to 200 °C represented the desorption of adsorbed water on the surface [35,36]; (2) mass loss observed from 200 to 500 °C was attributed to the removal of chemically combined water molecules or the pyrolysis of the loaded surfactant [37,38]; (3) mass loss from 500 to 800 °C was due to the dehydroxylation of structural OH units in the clays [39]. For NA, 0.97%, 1.57%, and 2.43% of mass loss was observed in the temperature region of 25–200 °C, 200–500 °C, and 500–800 °C, while the mass loss of OA was 0.54%, 7.71%, and 1.93%, respectively (Table 3). Thus, it can be seen that the mass loss of OA decreased from 25 to 200 °C and increased significantly from 200 °C to 500 °C, accounting for about 75.7% of total mass loss, confirming that the hydrophobicity of OA surface increased due to the presence of the surfactant. The DSC curves of NA and OA differed slightly (Figure 6b). NA and OA have three identical endothermic peaks at about 152 °C, 571 °C, and 658 °C, respectively. In addition, there was a special endothermic peak in OA at 251 °C, which was attributed to the oxidation of surfactants [35].

### 2.2. Adsorption Behaviors for ZEN

**Figure 7:**
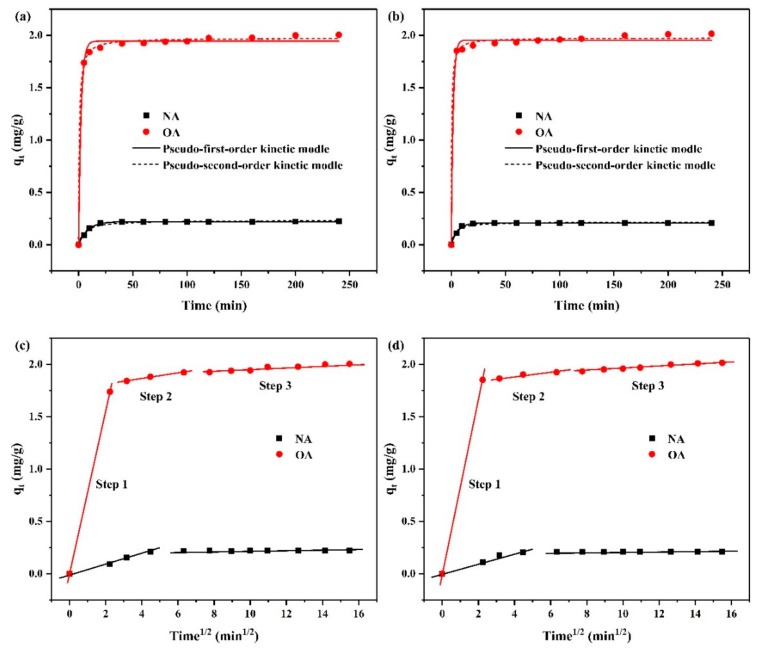
Kinetic curves of ZEN on natural albite (NA) and the prepared organoalbite (OA) at pH 7 (**a**,**c**), and pH 3 (**b**,**d**).

Figure 7 shows the changes of ZEN adsorption rate on NA and OA over time at pH 7 and pH 3. NA exhibited the same adsorption curves at pH 7 and pH 3. Most of the ZEN adsorption was completed in 20 min; afterward, the curves leveled off. For OA, the adsorption amount and adsorption rate of ZEN were much larger than NA. Owing to the presence of large numbers of adsorption sites, the rapid adsorption of ZEN occurred in the first 5 min. Subsequently, the effective adsorption sites gradually decreased with the prolongation of contact time, resulting in a slow increase in adsorption capacity, and reached equilibrium in 40 min. Adsorption kinetics can reveal the interaction mechanism in the adsorption process [40]. Based on the experimental data, three typical adsorption kinetic models, including the pseudo-first-order model, pseudo-second-order model, and the intraparticle diffusion model, were used to further explore the mechanism of the adsorption process of ZEN on NA and OA [41,42]. 

The expression of the pseudo-first-order model is given as follows:

(1) $$ln\left(q_{e}-q_{t}\right)=lnq_{e}-k_{1}t$$

The expression of the pseudo-second-order model is given as follows:

(2) $$\frac{t}{q_{t}}=\frac{1}{k_{2}q_{e}{}^{2}}+\frac{t}{q_{e}}$$

The expression of the intraparticle diffusion model is given as follows:

(3) $$q_{t}=k_{id}t^{\frac{1}{2}}+c$$

where q_e_ (mg/g) and q_t_ (mg/g) are the amount of ZEN adsorbed on adsorbent at equilibrium and at the sampling time t (min), while k_1_ (1/min), k_2_ (g/mg/min), and k_id_ (mg/g/min^0.5^) represent the equilibrium rate constants of the pseudo-first-order model, pseudo-second-order model, and intraparticle diffusion model, respectively. c is a constant related to the surface layer thickness.

Figure 7a,b and Table 4 show the fitting parameters of pseudo-first-order kinetic and pseudo-second-order kinetic. Compared to NA, the equilibrium adsorption values of OA for ZEN were up to 8.84-fold and 9.35-fold at pH 7 and pH 3, respectively. For NA, the higher R^2^ values were observed for the pseudo-first-order kinetic model at both of pH 7 and pH 3, which demonstrates that the ZEN adsorption was controlled by diffusion. However, for OA, the experimental data were fitted slightly better by the pseudo-second-order model than the pseudo-first-order model at both pH 7 and pH 3, indicating that chemisorption was the rate-limiting step during this adsorption process [43]. Generally, adsorption processes in porous solids can be separated into three steps: (1) Transfer from the bulk solution to the external surface of the sorbent (film diffusion); (2) diffusion from the surface into the pores (pore diffusion); and (3) adsorption equilibrium due to saturation of diffusion [40,44]. Figure 7c,d shows the multilinear plots of the intraparticle diffusion process of ZEN adsorption onto NA and OA. Three defined stages took place during the ZEN adsorption process on OA, and the capture of ZEN by NA included the first stage and the third stage. The fitting lines of the second and third stages did not pass through the origin, disclosing that the pore diffusion was not the rate-limiting step during the entire process [45]. Therefore, it could be inferred that ZEN adsorption onto NA and OA was dominated by the external and internal diffusion together. Moreover, no significant difference in adsorption kinetics was observed in the two different pH environments.

### 2.3. Adsorption Isotherm

**Figure 8:**
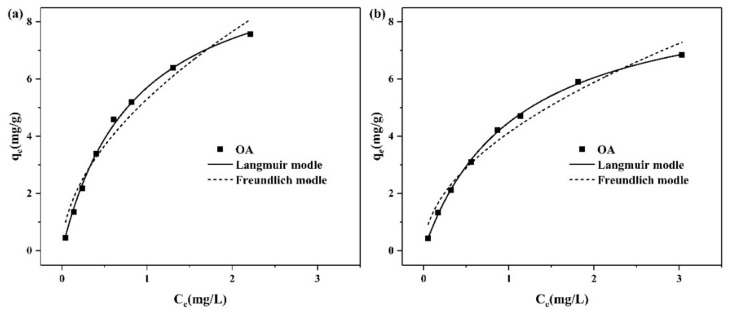
Kinetic curves of ZEN on the prepared organoalbite (OA) at pH 7 (**a**) and pH 3 (**b**).

The adsorption isotherm is frequently applied to elucidate equilibrium adsorption experimental data. We just showed the adsorption isotherm of OA because NA only adsorbed very little ZEN due to its hydrophilic nature and strong polarity. Figure 8a,b reveals that the adsorption capacity of OA increased with the increase of ZEN concentration and gradually reached saturation. The Langmuir and Freundlich models were used to fit the equilibrium isotherm data. The Langmuir isotherm assumes that the adsorbate uniformly adsorbs to the surface of adsorbent to form a monolayer, which indicates that no further adsorption occurs once the specific sites of the adsorbent are filled [18]. The Langmuir equation is expressed as follows:

(4) $$\frac{c_{e}}{q_{e}}=\frac{1}{k_{L}q_{m}}+\frac{c_{e}}{q_{m}}$$

where q_e_ (mg/g) is the amount of ZEN adsorbed at equilibrium, c_e_ (mg/L) represents the equilibrium concentration, k_L_ (L/mg) is the Langmuir constant, and q_m_ (mg/g) represents the maximum adsorption capacity.

The derivation of the Freundlich model assumes multilayer adsorption on the heterogeneous surface [18]. The Freundlich equation is expressed as follows:

(5) $$\ln q_{e}=lnk_{F}+\frac{1}{n}lnc_{e}$$

where k_F_ is the constant of the Freundlich isotherm, 1/n is the adsorption intensity exponent, and the other parameters are the same as those defined as in Equation (4).

Both the fitting curve and correlation coefficient (R^2^) show that the adsorptions of ZEN by OA at pH 7 and pH 3 were better fitted to the Langmuir model, indicating monolayer adsorption. In Table 5, the maximum adsorption capacity of OA was 10.580 mg/g at pH 7, which was larger than the uptake capacity of OA at pH 3 (9.287 mg/g). ZEN is a diphenol compound with an estimated pK_a1_ of 7.62, suggesting that it is mainly neutral at pH 3, while the phenolate anion is present in solution at pH 7 [25]. The increase in adsorption at pH 7 suggests that the interaction between ZEN and the positive portion of the CPC ions helped to increase adsorption. Characterization results show that the hydrophobicity of NA increased after modification, which improved the affinity of OA to the hydrophobic, weakly polar ZEN molecules, since the surface loading of CPC on NA acts as a partition medium. The adsorption ability was quickly enhanced with the increase in ZEN at low concentration, which could be due to the unsaturated active sites. With the further increase of concentration, the adsorption sites were saturated, and the physical adsorption contributed to the further improvement of adsorption capacity. This result indicates that the efficient active sites on OA were limited.

### 2.4. Desorption

**Figure 9:**
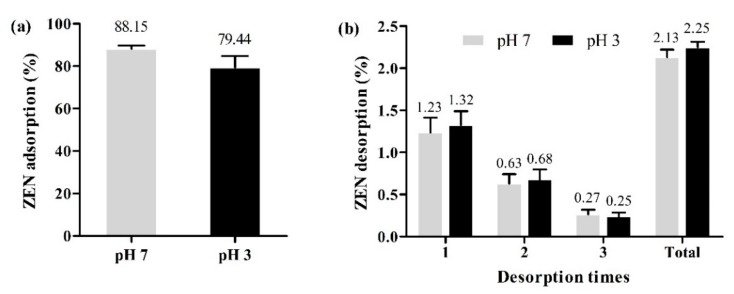
Adsorption (**a**) and desorption (**b**) of ZEN by OA at pH 7 and pH 3 (temperature = 37 °C; contact time = 60 min; adsorbent dose = 1.0 g/L, initial ZEN concentration = 5 mg/L; desorption time = 60 min).

To investigate the adsorption stability of ZEN by OA, the desorption experiments were repeated three times after the adsorption experiment. It is known that pH plays an important role in the adsorption of mycotoxins. As depicted in Figure 9a, the adsorption capacity in pH of 7 condition (88.15%) was higher than that in pH of 3.5 condition (79.44%), which indicates that further adsorption of OA would occur from the stomach to the intestine. This may be due to the influence of solution pH on cations on the surface of OA. The results of ZEN desorption (Figure 9b) showed that with the increasing desorption time the desorption rate decreased from 1.23 to 0.27% at pH 7 and from 1.32 to 0.25% at pH 3, respectively. After three desorption experiments, the total desorption rates of OA were very low and showed no difference at two pH values (2.13% at pH 7 and 2.25% and pH 3). These results demonstrate that OA has high adsorption ability and low desorption rate for ZEN in the gastrointestinal tract and has great potential in real-world application.

### 2.5. Effect of Co-Existing Amino Acids, Vitamins and Minerals

**Figure 10:**
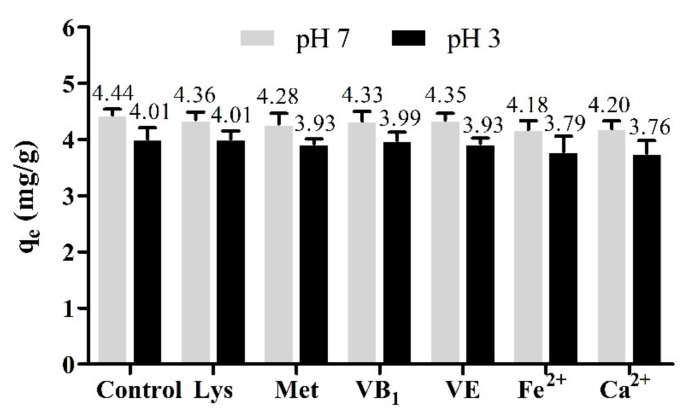
Effect of co-existing amino acids, minerals, and vitamins on the adsorption performance of OA for ZEN (temperature = 37 °C; contact time = 60 min; adsorbent dose = 1.0 g/L, initial ZEN concentration = 5 mg/L; Lys concentration = 20 mg/mL; Met concentration = 10 mg/mL; VB_1_ concentration = 4 mg/L; VE concentration = 50 IU/L; Fe^2+^ concentration = 5 mmol/L; Ca^2+^ concentration = 0.1 mol/L).

Amino acids, vitamins, and minerals are important nutrients in feed, which coexist with mycotoxins in contaminated feed. So, the effect of co-existing amino acids (i.e., Lys and Met), vitamins (i.e., VB_1_ and VE), and minerals (i.e., Fe^2+^ and Ca^2+^) on the adsorption of ZEN by OA was studied, and the results are shown in Figure 10. The removal efficiency of ZEN was not affected in the presence of Lys, Met, VB_1_, and VE, although decreased slightly in the presence of Fe^2+^ and Ca^2+^ at both of pH 7 and pH 3. These results demonstrate that OA had good adsorption selectivity for ZEN and was not affected by co-existing amino acids, vitamins, and minerals. 

## 3. Conclusions

In the present study, a novel organoalbite was prepared and applied for the detoxification of weakly polar ZEN from simulated gastrointestinal tract. The characterization results indicate that the loading of organic cationic surfactants increased the organic carbon content and converted the surface of NA from hydrophilic to hydrophobic, which facilitated the removal of ZEN. Compared with NA, OA showed higher adsorption capacity for ZEN (10.580 mg/g at pH 7 and 9.287 mg/g at pH 3). The kinetics of ZEN adsorption by OA was well simulated by the pseudo-second-order model. In addition, the internal particle diffusion model showed that ZEN adsorption was dominated by both external and internal diffusion. The Langmuir isotherm model was confirmed to be more suitable for describing the ZEN adsorption behavior of OA than the Freundlich model, indicating that the adsorption process occurred as monolayer adsorption on the layer surface of the OA structure. Moreover, OA had good adsorption stability and selectivity. The desorption rate of ZEN was only about 2% after three desorption experiments, and ZEN adsorption was not affected by co-existing amino acids, vitamins, or minerals. These results suggest that OA possesses tremendous potential as a mycotoxin adsorbent for the detoxification of the hydrophobic, weakly polar ZEN.

## 4. Materials and Methods

### 4.1. Materials

The natural ground albite (75 μm) from Shijiazhuang (Hebei Province, China) was purified by centrifugal separation using sodium hexametaphosphate as a dispersant [46]. The cation exchange capacity (CEC) was 26.66 meq/100 g quantified by the ammonium chloride method [47]. Sodium hexametaphosphate ((NaPO_3_)6), CPC (C_21_H_38_ClN·H_2_O, 99%) and phosphoric acid (H_3_PO_4_, 85%) were purchased from Shanghai Macklin Biochemical Co., Ltd. (Shanghai, China). The ZEN standard, acetonitrile and methyl alcohol were obtained from Sigma-Aldrich (St. Louis, MO, USA). Phosphoric acid, acetonitrile and methyl alcohol were of chromatographic purity, and other reagents were of analytical purity. All aqueous solutions were prepared using deionized water.

### 4.2. Preparation of Organoalbite

Natural albite was modified by cation exchange with the cationic surfactant cetylpyridinium chloride (CPC). A stoichiometric amount (100% of CEC) of CPC and 2 g of NA were added in 200 mL of deionized water in turn, and then the mixture was shaken (200 rpm) for 1 h at 50 °C in a shaker bath. Next, the prepared OA was filtered and washed with deionized water until there was no CPC residue in the liquid (tested by AgNO_3_). Finally, it was dried in an oven at 60 °C and ground to a particle size of less than 75 μm.

### 4.3. Characterization

Field-emission SEM (S-3400N, Hitachi, Tokyo, Japan) was applied to observe the surface morphologies of albite before and after modification. The XRD analysis was carried out on a Bruker D8 Advance diffractometer (Bruker AXS, Germany) with a Cu Kα radiation (λ = 0.15406 nm) operating at 40 kV and 20 mA. Scans were made at room temperature from 5 to 50 °C with a step of 2 °C/min. Elemental analyses of C, H, and N were conducted on an Elementar (Vario III) system (Elementar, Germany). The surface functional groups were observed by FTIR spectra in the 4000–400 cm^−1^ range with a spectral resolution of 1 cm^−1^ on a Nicolet Nexus 410 FTIR spectrometer (Nicolet, USA) using the KBr pellets. The thermal analysis data were collected using a Netzsch STA 449 F3 thermo analyzer (Netzsch, Germany), heating in the temperature range of 25 to 800 °C with a heating ramp of 10 °C/min under an N_2_ atmosphere. Moreover, the hydrophobicity of NA and OA were measured from the moisture adsorption experiments, which were carried out at 20 °C and 33% relative humidity. All samples were dried at 105 °C for 24 h before the tests were conducted. According to the change in mass of the samples, the moisture adsorption capacities were calculated by the following equation:

(6) $$Ma=\frac{m_{t}-m_{0}}{m_{0}}\times 100\%$$

where Ma is moisture content, m_0_ is the initial weight of the dried sample, and m_t_ is the weight of the sample at time t.

N_2_ adsorption–desorption isotherms were detected on a Micromeritics ASAP 2460 instrument (Micromeritics Inc., USA) at 77 K. The pore structure parameters such as surface area, pore volume, and average pore size were calculated by the BET and Barrett–Joyner–Halenda (BJH) methods. The samples were heated at 105 °C for 12 h prior to N_2_ adsorption.

### 4.4. Batch Adsorption Experiments

pH 3 is equivalent to stomach and pH 7 is equivalent to intestine conditions [48]. The mycotoxin adsorption experiments were conducted in phosphate buffer solutions, which simulated the pH conditions in the gastrointestinal tract. Stock ZEN solution (100 mg/L) was prepared by dissolving ZEN powder in acetonitrile and then diluting to different concentrations with 0.1 M phosphate buffer (pH 3 and 7) for further use. The pH was adjusted using H_3_PO_4_ or NaOH. To study the adsorption kinetics of ZEN, 10 mg of NA or OA was added into 10 mL of the 2 mg/L ZEN solution. Then, the mixture was placed on a rotary shaker and shaken at 200 rpm at 37 °C. Samples were withdrawn at 5, 10, 20, 40, 60, 80, 100, 120, 160, 200, and 240 min. To study the adsorption isotherms of ZEN, 10 mg of NA or OA was added into 10 mL of ZEN solution (0.5–10.0 mg/L). The experiments were conducted for 60 min. The desorption experiments were carried out after the adsorption determination of 10 mg of OA and 10 mL of ZEN solution (5.0 mg/L). Ten milliliters of phosphate buffer was added to the remaining OA after supernatant removal, then the mixture was shaken at 37 °C for 60 min. The experiments were repeated three times. To investigate the selective adsorption of OA, 10 mg of OA was immersed in 10 mL of 5 mg/L ZEN mixture containing lysine (Lys, 20 mg/mL), methionine (Met, 10 mg/mL), vitamin B_1_ (VB_1_, 4 mg/L), vitamin E (VE, 50 IU/L), Fe^2+^ (5 mmol/L), or Ca^2+^ (0.1 mol/L), respectively. All mixtures were centrifuged for 5 min and filtered with a 0.22 μm filter membrane for analysis of ZEN concentration by HPLC. In addition, a control treatment without adsorbent was carried out for each experiment. 

The adsorption properties of albite before and after modification with respect to ZEN were studied by analyzing ZEN concentration changes in the reaction solution. The ZEN concentrations were analyzed using an immunoaffinity column purification technique and determined by HPLC. The Waters HPLC system was equipped with a binary pump (model 600), a fluorescence detector (model 2475 multi λ, λex = 274 nm and λem = 440 nm), and an autosampler (model 2707). A reversed-phase C18 column (Zorbax SB, 250 × 4.6 mm, 5 μm i.d.; Agilent Technologies Inc., Palo Alto, CA, USA) was used to separate sample constituents. The mobile phase containing an acetonitrile/methanol/water (46:8:46, v/v/v) solution was used with a flow rate of 1 mL/min. The injection volume was 20 μL. The amount of adsorbed ZEN was calculated using the following equation:

(7) $$q_{e}=\frac{c_{0}-c_{t}}{m}\times V$$

where c_0_ (mg/L) is the initial concentration of ZEN, c_t_ (mg/L) is the concentration of ZEN at time t, V (L) is the volume of solution used, and m (g) is the mass of the NA or OA sample. A calibration curve was plotted with peak area as a function of ZEN concentration in solution.

## Figures and Tables

**Table 1:** Organic elemental analysis of natural albite (NA) and the prepared organoalbite (OA).

Sample	C (w%)	H (w%)	N (w%)	GSL (CEC)
NA	-	-	-	0.000
OA	5.94	1.26	0.34	0.998

GSL: Genuine surfactant loading, calculated from N analysis; CEC, cation exchange capacity.

**Table 2:** Surface parameters of natural albite (NA) and the prepared organoalbite (OA).

Sample	S_BET_^a^ (m^2^/g)	Pore Volume ^b^ (cm^3^/g)	Pore Size ^c^ (nm)
NA	9.80	0.02	7.92
OA	4.87	0.02	17.86

^a^ S_BET_ means specific surface areas calculated using BET theory. ^b^ Pore volume refers to BJH desorption cumulative volume of pores between 2.0192 and 300 nm in diameter. ^c^ Pore size is the BJH desorption average pore size (4 V/A).

**Table 3:** Mass loss of natural albite (NA) and the prepared organoalbite (OA) in different temperature regions.

Sample	Mass Loss (%)			
	25–200 °C	200–500 °C	500–800 °C	∑(25–800) °C
NA	0.97	1.57	2.43	4.97
OA	0.54	7.71	1.93	10.18

**Table 4:** Adsorption kinetic parameters for ZEN on natural albite (NA) and the prepared organoalbite (OA).

Kinetic Model	Parameters	pH = 7		pH = 3	
		NA	OA	NA	OA
Pseudo-first order	q_e_ (mg/g)	0.220	1.945	0.209	1.954
	k_1_ (1/min)	0.120	0.431	0.166	0.573
	R^2^	0.997	0.994	0.997	0.993
Pseudo-second order	q_e_ (mg/g)	0.233	1.975	0.218	1.975
	k_2_ (g/mg/min)	0.812	0.701	1.372	1.202
	R^2^	0.974	0.998	0.974	0.997

**Table 5:** Isotherm parameters for ZEN adsorption by organoalbite (OA).

pH	Langmuir Model			Freundlich Model		
	q_m_ (mg/g)	k_L_ (L/mg)	R^2^	k_F_ (mg^1 − 1/n^ L^1/n^/g)	1/n	R^2^
pH 7	10.580	1.171	0.998	5.301	0.531	0.960
pH 3	9.287	0.935	0.999	4.131	0.512	0.970
